# Supplementary material for: Sugar Metabolism of the First Thermophilic Planctomycete Thermogutta terrifontis: Comparative Genomic and Transcriptomic Approaches
Source: Front Microbiol. 2017 Nov 2;8:2140. doi: 10.3389/fmicb.2017.02140 (PMC5673643; doi:10.3389/fmicb.2017.02140)
Supplement: Supplementary file 2 [file Data_Sheet_2.DOCX]

Supplementary Table 2. *T. terrifontis* R1 glycosidases, polysaccaride lyases and

carbohydrate esterases.

| **Gene, *THTE_*** | **Function** | **Domains** | **SignalP** | **SecretomeP** | **LipoP (SpII results only)** | **TMHMM** |
| --- | --- | --- | --- | --- | --- | --- |
| 208 | probable beta-galactosidase or beta-glucuronidase or new activity in GH2 | GH2+CBM67 | - | - | - | 0 |
| 396 | put GH | - | - | + | - | 0 |
| 436 | probable polygalacturonase | GH28 | + | - | - | 0 |
| 474 | put GH | CBM66 | - | - | - | 1 |
| 494 | Alpha-L-fucosidase | GH29 | + | - | - | 0 |
| 655 | put GH | CBM4+GH51 | - | - | - | 0 |
| 688 | probable beta-xylosidase | GH39 | - | - | - | 0 |
| 696 | probable fructosidase | CBM38+GH32 | - | - | - | 0 |
| 808 | put PL | PL12 | - | - | - | 0 |
| 842 | put GH | - | - | - | - | 0 |
| 861 | put PL | - | - | - | - | 0 |
| 880 | put GH | - | + | + | - | 1 |
| 890 | endoglucanase | GH5 | - | - | - | 0 |
| 913 | probable alpha-L-rhamnosidase | GH106 | - | - | - | 0 |
| 963 | GH116 | CBM38+GH116 | - | - | - | 0 |
| 1038 | put GH | CBM66 | + | + | - | 0 |
| 1077 | put PL | - | - | - | - | 1 |
| 1080 | probable alpha-L-rhamnosidase | CBM32+GH106 | - | - | - | 0 |
| 1086 | beta-L-arabinofuranosidase | GH127 | - | - | - | 0 |
| 1171 | GH5 | GH5 | + | - | - | 0 |
| 1180 | put GH | CBM51 | - | - | - | 1 |
| 1217 | probable sialidase | GH33+GH93 | - | - | - | 0 |
| 1226 | probable alpha-L-rhamnosidase | GH106 | + | - | - | 0 |
| 1288 | put PL | PL15 | - | - | - | 1 |
| 1430 | probable alpha-L-rhamnosidase | GH106+CBM67 | - | - | - | 0 |
| 1440 | probable alginate lyase | PL15 | - | + | - | 0 |
| 1454 | put GH | - | - | - | + | 0 |
| 1477 | Isoamylase | GH13 | - | - | - | 0 |
| 1485 | beta-L-arabinofuranosidase | GH127 | - | - | - | 0 |
| 1488 | rhamnogalacturonan lyase | PL11 | - | - | - | 0 |
| 1516 | polygalacturonase | GH28 | - | - | - | 0 |
| 1560 | probable two-domain alpha-galactosidase | GH36+GH36 | + | - | - | 0 |
| 1561 | put GH | CBM66 | - | - | - | 1 |
| 1595 | endo-1,2-aplha-mannosidase | GH99 | - | - | - | 0 |
| 1596 | GH5 | GH5+CBM4 | - | - | - | 1 |
| 1652 | GH36 | GH36 | + | - | - | 0 |
| 1668 | probable alpha-L-arabinofuranosidase | CBM66+GH51 | - | - | - | 1 |
| 1726 | xylanase or acetylxylan esterase | CE10 | + | - | - | 1 |
| 1734 | GH5 | GH5 | - | - | - | 0 |
| 1774 | GH33 | GH33 | - | - | - | 1 |
| 1776 | probable beta-hexoseaminidase | GH20 | - | - | - | 0 |
| 1802 | put GH | CBM51 | - | - | - | 0 |
| 1819 | GH39 | CBM4+CBM16+GH39 | - | - | - | 1 |
| 1884 | probable beta-xylosidase | GH39 | - | - | - | 1 |
| 1929 | put GH | CBM16+GH20 | - | - | - | 0 |
| 1966 | put PL | CBM35 | - | + | - | 1 |
| 1993 | probable pectate lyase | PL10 | - | - | - | 0 |
| 2055 | probable sialidase | GH33 | - | + | - | 0 |
| 2056 | put PL | PL6 | - | - | - | 1 |
| 2101 | alpha-L-rhamnosidase | CBM67+CBM67+GH78 | + | - | - | 0 |
| 2104 | probable beta-galactosidase or beta-glucuronidase or new activity in GH2 | GH2 | + | - | - | 0 |
| 2108 | GH39 | GH39 | + | - | - | 0 |
| 2109 | probable alpha-galatosidase (GH27) | CBM51 | - | - | - | 1 |
| 2121 | probale polygalacturonase | GH28 | - | - | - | 0 |
| 2124 | probable beta-L-arabinosidase | GH127 | - | - | - | 0 |
| 2126 | probable GH5 or GH42 | GH5 | - | - | - | 1 |
| 2137 | probable endo-beta-1,4-mannosidase | GH26+CBM32 | - | - | - | 0 |
| 2143 | 4-alpha-glucanotransferase | GH77 | - | - | - | 0 |
| 2173 | put GH | GH99+CBM40 | - | - | - | 1 |
| 2177 | probable beta-L-arabinosidase | GH127 | - | - | - | 0 |
| 2188 | unsaturated glucuronyl hydrolase | GH88 | - | - | - | 0 |
| 2189 | alpha-L-arabionfuranosidase | GH51 | + | - | - | 1 |
| 2199 | put GH | GH5 | + | - | - | 0 |
| 2234 | put GH | GH33 | - | + | - | 0 |
| 2342 | probable heparin-sulfate lyase | PL12 | - | - | - | 0 |
| 2388 | GH42 | CBM4+GH42 | - | - | - | 0 |
| 2391 | put GH | GH28 | + | - | - | 0 |
| 2393 | put GH | GH42 (high E-value) | + | - | - | 0 |
| 2406 | probable alginate lyase (PL15) or PL12 | PL12 | - | - | - | 1 |
| 2430 | probable beta-glucosidase | GH116 | - | - | - | 0 |
| 2480 | put CE/GH | CE3+CBM66 | - | - | - | 1 |
| 2600 | put GH | GH10 | - | - | - | 0 |
| 2605 | probable alpha-mannosidase | GH38 | - | - | - | 0 |
| 2697 | GH123 | GH123 | - | - | - | 0 |
| 2787 | put GH | - | - | - | - | 0 |
| 2789 | sialidase | GH33 | - | + | - | 0 |
| 2820 | probable beta-galactosidase or beta-glucuronidase or new activity in GH2 | GH2+CBM32 | - | - | - | 0 |
| 2824 | acetylxylan esterase/cephalosporin-C deacetylase | CE7 | - | - | - | 0 |
| 2830 | put PL | - | - | + | - | 0 |
| 2852 | probable alpha-L-rhamnosidase | GH78 | + | - | - | 0 |
| 2853 | put GH | CBM66 | + | - | - | 0 |
| 2856 | exo-beta-agarase | GH50 | - | - | - | 0 |
| 2930 | put GH | GH93 | - | - | - | 0 |
| 3078 | probable beta-1,3-glucanase | GH81 | - | + | - | 0 |
| 3153 | 1,4-alpha-glucan branching enzyme | CBM48+CBM48+GH13 | - | - | - | 0 |
| 3216 | GH5 | GH5 | - | - | - | 1 |
| 3262 | probable alpha-agarase | GH96 | - | - | - | 0 |
| 3305 | alpha-L-arabionfuranosidase | GH62 | - | - | - | 0 |
| 3333 | GH5 | CBM35+GH5 | + | - | + | 0 |
| 3338 | put GH | CBM66 | - | - | - | 0 |
| 3372 | GH5 | GH5 | - | - | - | 0 |
| 3386 | GH5 | GH5 | + | - | - | 0 |
| 3432 | probable beta-1,3-glucanase or beta-porphyranase | GH16 | - | + | - | 0 |
| 3433 | probable beta-galactosidase or beta-glucuronidase or new activity in GH2 | GH2 | - | - | - | 0 |
| 3534 | put GH | - | - | + | - | 1 |
| 3544 | put GH | - | - | - | - | 0 |
| 3520 | put GH | GH38 | - | - | - | 0 |
| 3553 | put PL/GH | - | - | + | - | 1 |
| 3584 | put GH | GH129 | - | - | - | 1 |
| 3601 | rhamnogalacturonan lyase | PL11 | - | + | - | 0 |
| 3688 | endoglucanase | GH5 | - | - | - | 0 |
| 3737 | put GH | - | - | - | - | 0 |
| 3783 | alpha-amylase or 4-alpha-glucano transferase | GH57 | - | - | - | 0 |
| 3787 | GH5 | GH5 | - | - | - | 0 |
| 3824 | probable beta-galactosidase or beta-glucuronidase or new activity in GH2 | GH2+CBM22+CBM4 | - | - | + | 1 |
| 3910 | put GH | GH43 | - | - | - | 0 |
| 3961 | endo-1,4-beta-xylanase | GH10 | - | - | - | 0 |
| 4088 | put GH | CBM9 | - | - | - | 0 |
| 4089 | put GH | - | + | - | - | 0 |
| 4098 | put PL | - | - | - | - | 0 |
| 4145 | N-acetylgalactosaminidase | GH129 | - | - | - | 1 |
| 4163 | put GH | - | + | - | - | 0 |
| 4199 | alpha-L-fucosidase | GH29+CBM32 | - | - | - | 0 |
| 4222 | put GH | GH76 | - | - | - | 0 |
| 4234 | put GH | GH32 | - | - | - | 0 |
| 4327 | GH99 | GH99 | - | - | - | 0 |
| 4390 | alpha-L-rhamnosidase | GH106+CBM67 | - | - | - | 1 |
| 4455 | put GH | - | - | + | - | 0 |

Supplementary Table 3. Embden-Meyerhof pathway (glycolysis) genes

| **Enzyme** | **Gene** |
| --- | --- |
| Glucokinase | *THTE_0095*, *THTE_2175*, *THTE_3207*, *THTE_4164* |
| Glucose-6-phosphate isomerase | *THTE_1855* |
| Phosphofructokinase | *THTE_0093*^1^, *THTE_1056*^1^, *THTE_2190*^2^, *THTE_2629*^1^ |
| Fructose-1,6-bisphosphate aldolase | *THTE_1419* |
| Glyceraldehyde-3- phosphate dehydrogenase | *THTE_2985* |
| Phosphoglycerate kinase | *THTE_3374* |
| Phosphglycerate mutase | *THTE_0470*, *THTE_1181* |
| Enolase | *THTE_1986* |
| Pyruvate kinase | *THTE_3477* |

^1^ – pyrophosphate (PPi)-dependent (EC 2.7.1.90).

^2^ – ATP-dependent (EC 2.7.1.11).

Supplementary Table 4. Pentose-5-phosphate pathway genes

| **Enzyme** | **Gene** |
| --- | --- |
| Glucose-6-P dehydrogenase | *THTE_1713* |
| 6-phosphogluconolactonase | *THTE_4372* |
| 6-phosphogluconate dehydrogenase | *THTE_2631* |
| Ribulose-5-P isomerase | *THTE_1333* |
| Ribulose-5-P epimerase | *THTE_0930* |
| Transketolase | *THTE_0645* |
| Transaldolase | No |
| PPi- phosphofructokinase (S-7-P phosphorylating) | *THTE_0093*, *THTE_2629* |
| Fructose-1,6-bisphosphate aldolase | *THTE_1419* |

Supplementary Table 5. Tricarboxylic acid cycle genes

| **Enzyme** | **Gene** |
| --- | --- |
| Citrate synthase | *THTE_1896* |
| Aconitase | *THTE_3127* |
| Isocitrate dehydrogenase | *THTE_1950* |
| Fd-dependent 2-oxoglutarate oxidoreductase | *THTE_0056* (alpha-subunit),  *THTE_0055* (beta-subunit) |
| Succinyl-CoA ligase | *THTE_3900* (alpha-subunit), *THTE_3898* (beta-subunit) |
| Succinate dehydrogenase | *THTE_3868* (FeS-subunit),  *THTE_3869* (flavoprotein subunit) |
| Fumarate hydratase | *THTE_0765* |
| Malate dehydrogenase | *THTE_1184* |

Supplementary Table 6. Aerobic electron transfer chain genes

| **Complex** | **Subunit** | **Protein** |
| --- | --- | --- |
| I (NADH-dehydrogenase) | A | THTE_0767 |
|  | B | THTE_0768 |
|  | C | THTE_0769 |
|  | D | THTE_0770 |
|  | E | THTE_0771 |
|  | F | THTE_0772 |
|  | G | THTE_0773 |
|  | H | THTE_0775 |
|  | I | THTE_0776 |
|  | J | THTE_0777 |
|  | K | THTE_0778 |
|  | L | THTE_0779 |
|  | M | THTE_0781 |
|  | N | THTE_0782 |
| II (Succinate dehydrogenase) | SdhA | THTE_3869 |
|  | SdhB | THTE_3868 |
|  | SdhC | THTE_3870 |
| III (b/c_1_ complex) | Cytochrome c_1_ | THTE_1510 |
|  | Cytochrome b | THTE_1511 |
|  | Rieske protein | THTE_1512 |
| IV (Cytochrome c oxidase aa_3_-type) | Subunit I | THTE_2904 |
|  | Subunit II | THTE_2903 |
|  | Subunit III | THTE_2907 |
|  | Subunit IV | THTE_2908 |

Supplementary Table 7. Distribution of genes, encoding proteins with DUF1080 (pfam06439) domains among bacterial genomes present in the IMG database*.

| **SEQUENCE STATUS** | **GENOME** | **PFAM06439** |
| --- | --- | --- |
| **TOP 30 GENOMES WITH THE HIGHEST NUMBER OF DETECTED DUF1080 CODING SEQUENCES** | | |
| P | *Verrucomicrobia* bacterium SCGC AAA164-O14 (unscreened) (genbank_version) | 38 |
| **P** | ***Rhodopirellula* sp. SWK7** | **36** |
| **P** | ***Rhodopirellula maiorica* SM1** | **33** |
| P | *Verrucomicrobia* bacterium SCGC AAA168-F10 | 33 |
| P | unclassified *Planctomycetaceae* Bin 56 | 31 |
| F | *Candidatus* Solibacter usitatus Ellin6076 | 31 |
| P | *Verrucomicrobiaceae* bacterium EBPR_Bin_287 | 30 |
| **P** | ***Roseimaritima ulvae* DSM 25454** | **29** |
| **P** | **Porphyra umbilicalis P.um.1-endophyte10645 P1 (Porphyra_umbilicalis_P.um.1-endophyte10645)** | **29** |
| **P** | ***Gimesia maris* DSM 8797** | **28** |
| **P** | ***Zavarzinella formosa* DSM 19928** | **28** |
| P | *Prosthecobacter debontii* ATCC 700200 | 28 |
| **D** | ***Gimesia maris* PLM2** | **28** |
| P | *Verrucomicrobium* sp. BvORR106 | 28 |
| P | *Verrucomicrobium spinosum* DSM 4136 | 27 |
| **P** | ***Rhodopirellula baltica* SH28** | **27** |
| **P** | ***Rhodopirellula baltica* SWK14** | **27** |
| **P** | ***Singulisphaera acidiphila* DSM 18658** | **27** |
| **P** | ***Rhodopirellula lusitana* DSM 25457** | **27** |
| **P** | ***Rhodopirellula baltica* WH47** | **27** |
| **P** | ***Rhodopirellula europaea* 6C** | **27** |
| **F** | ***Planctomyces* sp. SH-PL14** | **27** |
| **F** | ***Singulisphaera acidiphila* MOB10, DSM 18658** | **27** |
| P | *Verrucomicrobia* bacterium SCGC AAA164-E04 (unscreened) | 27 |
| P | unclassified *Verrucomicrobiales* Bin 46 | 27 |
| P | *Arenibacter algicola* TG409 | 27 |
| **P** | **Porphyra umbilicalis P.um.1-endophyte06694 P2 (Porphyra_umbilicalis_P.um.1-endophyte06694)** | **27** |
| P | *Mariniphaga anaerophila* DSM 26910 | 26 |
| **P** | ***Rhodopirellula europaea* SH398** | **26** |
| **P** | ***Singulisphaera* sp. GP187** | **26** |
| **OTHER PLANCTOMYCETES GENOMES** | | |
| **F** | ***Rhodopirellula baltica* SH 1** | **25** |
| **D** | ***Planctomycetes* bacterium RBG_16_64_12** | **25** |
| **P** | ***Rhodopirellula islandica* K833** | **25** |
| **P** | ***Blastopirellula marina* SH 106T, DSM 3645** | **25** |
| **F** | ***Rubinisphaera brasiliensis* DSM 5305** | **24** |
| **P** | ***Schlesneria paludicola* DSM 18645** | **23** |
| **P** | ***Planctomicrobium piriforme* DSM 26348** | **22** |
| **P** | ***Planctomycetes* bacterium SM23_25** | **22** |
| **P** | ***Gemmata obscuriglobus* UQM 2246** | **22** |
| **D** | ***Planctomycetes* bacterium RBG_16_55_9** | **22** |
| **P** | **unclassified *Planctomycetaceae* Bin 22** | **20** |
| **D** | ***Pirellula* sp. Pr1d** | **20** |
| **P** | ***Phycisphaerae* bacterium SG8_4** | **20** |
| **F** | ***Planctomyces* sp. SH-PL62** | **20** |
| **D** | **Composite genome from Lake Mendota Epilimnion pan-assembly MEint.metabat.15954** | **20** |
| **P** | ***Gemmata massiliana* IIL30** | **20** |
| **F** | ***Planctopirus limnophila* DSM 3776** | **20** |
| **P** | ***Planctomycetaceae* bacterium FC18** | **20** |
| **F** | ***Pirellula staleyi* DSM 6068** | **19** |
| **D** | **Composite genome from Lake Mendota Epilimnion pan-assembly MEint.metabat.3611** | **19** |
| **F** | ***Gemmata* sp. SH-PL17** | **19** |
| **D** | **Composite genome from Lake Mendota Epilimnion pan-assembly MEint.metabat.3061** | **18** |
| **P** | ***Planctomycetales* bacterium JGI 01_E22 (unscreened)** | **18** |
| **F** | ***Pirellula* sp. SH-Sr6A** | **18** |
| **D** | ***Planctomycetes* bacterium RBG_13_63_9** | **17** |
| **D** | **Composite genome from Lake Mendota Epilimnion pan-assembly MEint.metabat.4620** | **16** |
| **D** | **Composite genome from Lake Mendota Epilimnion pan-assembly MEint.metabat.9166** | **16** |
| **P** | **unclassified *Planctomycetaceae* Bin 32** | **15** |
| **D** | **Composite genome from Lake Mendota Epilimnion pan-assembly MEint.metabat.20494** | **15** |
| **P** | ***Planctomycetales* bacterium JGI 01_G17 (unscreened)** | **14** |
| **P** | ***Planctomycetales* bacterium JGI 01_G17 (contamination screened)** | **14** |
| **D** | **Composite genome from Lake Mendota Epilimnion pan-assembly MEint.metabat.2907** | **14** |
| **D** | **Composite genome from Lake Mendota Epilimnion pan-assembly MEint.metabat.10020** | **14** |
| **D** | ***Planctomycetes* bacterium RBG_16_64_10** | **13** |
| **P** | ***Rubripirellula obstinata* LF1** | **13** |
| **D** | **Composite genome from Lake Mendota Epilimnion pan-assembly MEint.metabat.14003** | **13** |
| **F** | ***Isosphaera pallida* IS1B, ATCC 43644** | **12** |
| **D** | **Planctomycetes bacterium RBG_19FT_COMBO_48_8** | **12** |
| **D** | **Planctomycetes bacterium RBG_13_60_9** | **11** |
| **D** | ***Plantomycetaceae* sp. genome_bin_12** | **11** |
| **D** | **Composite genome from Lake Mendota Epilimnion pan-assembly MEint.metabat.7803** | **10** |
| **D** | ***Planctomycetes* bacterium RBG_13_62_9** | **10** |
| **P** | **Porphyra umbilicalis P.um.1-endophyte07873 (Porphyra_umbilicalis_P.um.1-endophyte07873)** | **9** |
| **D** | ***Planctomycetes* bacterium RBG_13_50_24** | **9** |
| **P** | ***Planctomycetes* bacterium DG_20** | **9** |
| **P** | **Kelp biofilm associated *Planctomycetes* bin 2 (Bin from Kelp Biofilm metagenome, processed and reassembled)** | **8** |
| **P** | **Kelp biofilm associated *Planctomycetes* bin 1 (Bin from Kelp Biofilm metagenome, processed and reassembled)** | **8** |
| **P** | ***Planctomycetaceae* bacterium JGI M3C4D3-002-D19 (unscreened)** | **8** |
| **P** | ***Phycisphaerae* bacterium SM1_79** | **7** |
| **P** | **unclassified *Planctomycetaceae* Bin 20** | **7** |
| **D** | **Composite genome from Lake Mendota Epilimnion pan-assembly MEint.metabat.18138** | **7** |
| **D** | **Composite genome from Lake Mendota Epilimnion pan-assembly MEint.metabat.12555** | **7** |
| **D** | **Composite genome from Lake Mendota Epilimnion pan-assembly MEint.metabat.7672** | **7** |
| **P** | **unclassified *Planctomycetaceae* bin 3 (Bin from Kelp Biofilm metagenome, processed and reassembled)** | **7** |
| **P** | ***Planctomycetaceae* bacterium JGI M3C4D3-002-D19 (contamination screened)** | **6** |
| **D** | ***Phycisphaerae* bacterium bin0** | **6** |
| **P** | **unclassified *Planctomycetaceae* bin 6 (Bin from Kelp Biofilm metagenome, processed and reassembled)** | **6** |
| **P** | ***Phycisphaerae* bacterium SM23_30** | **5** |
| **P** | ***Phycisphaeraceae* bacterium EBPR_Bin_183** | **5** |
| **P** | ***Phycisphaeraceae* bacterium EBPR_Bin_263** | **5** |
| **D** | ***Planctomycetes* bacterium RBG_13_44_8b** | **5** |
| **P** | **unclassified *Planctomycetaceae* Bin 56-2** | **4** |
| **P** | **unclassified *Planctomycetaceae* bin 5 (Bin from Kelp Biofilm metagenome, processed and reassembled)** | **4** |
| **P** | **unclassified *Planctomycetaceae* Bin 63-2** | **4** |
| **D** | ***Pirellula* sp. Pir 4d** | **4** |
| **P** | **unclassified *Planctomycetaceae* Bin 63** | **3** |
| **P** | **unclassified *Planctomycetaceae* Bin 56-1** | **3** |
| **P** | **unclassified *Planctomycetaceae* bin 8 (Bin from Kelp Biofilm metagenome, processed and reassembled)** | **3** |
| **D** | ***Planctomycetes* bacterium RBG_13_46_10** | **2** |
| **D** | ***Planctomycetes* bacterium JGI 0000014-I13 (unscreened)** | **2** |
| **P** | **unclassified *Planctomycetaceae* Bin 63-1** | **2** |
| **P** | **unclassified *Planctomycetaceae* bin 7 (Bin from Kelp Biofilm metagenome, processed and reassembled)** | **2** |
| **D** | ***Planctomycetes* bacterium SCGC JGI090-P21 (unscreened)** | **2** |
| **P** | ***Candidatus* Brocadia sinica JPN1** | **1** |
| **P** | ***Planctomycetes* bacterium SM23_32** | **1** |
| **P** | ***Planctomycetes* bacterium JGI F02006-K19 (contamination screened)** | **1** |
| **P** | ***Planctomycetes* bacterium RIFCSPHIGHO2_02_FULL_40_12** | **1** |
| **P** | ***Planctomycetes* bacterium JGI B05045-H15 (contamination screened)** | **1** |
| **P** | ***Planctomycetes* bacterium RIFOXYD2_FULL_41_16** | **1** |
| **P** | ***Planctomycetes* bacterium SM23_65** | **1** |
| **D** | ***Planctomycetes* bacterium GWA2_39_15** | **1** |
| **P** | **Candidatus Kuenenia stuttgartiensis** | **0** |
| **P** | **Candidatus Kuenenia stuttgartiensis CH1** | **0** |
| **P** | **Candidatus Kuenenia stuttgartiensis RU1** | **0** |
| **F** | **Phycisphaera mikurensis NBRC 102666** | **0** |
| **P** | **Planctomycetaceae bacterium KSU-1** | **0** |
| **D** | **Planctomycetes bacterium SCGC AAA257-G04 (unscreened)** | **0** |
| **D** | **Planctomycetes bacterium SCGC JGI014-B07 (unscreened)** | **0** |
| **P** | **Planctomycetia bacterium EBPR_Bin_174** | **0** |

*- The analysis was done 31.05. 2017. *Planctomycetes* representatives are in bold. Representatives of *Planctomycetia* class are highlighted by light gray color. Genome sequence status: D – draft, P – permanent draft, F – finished.


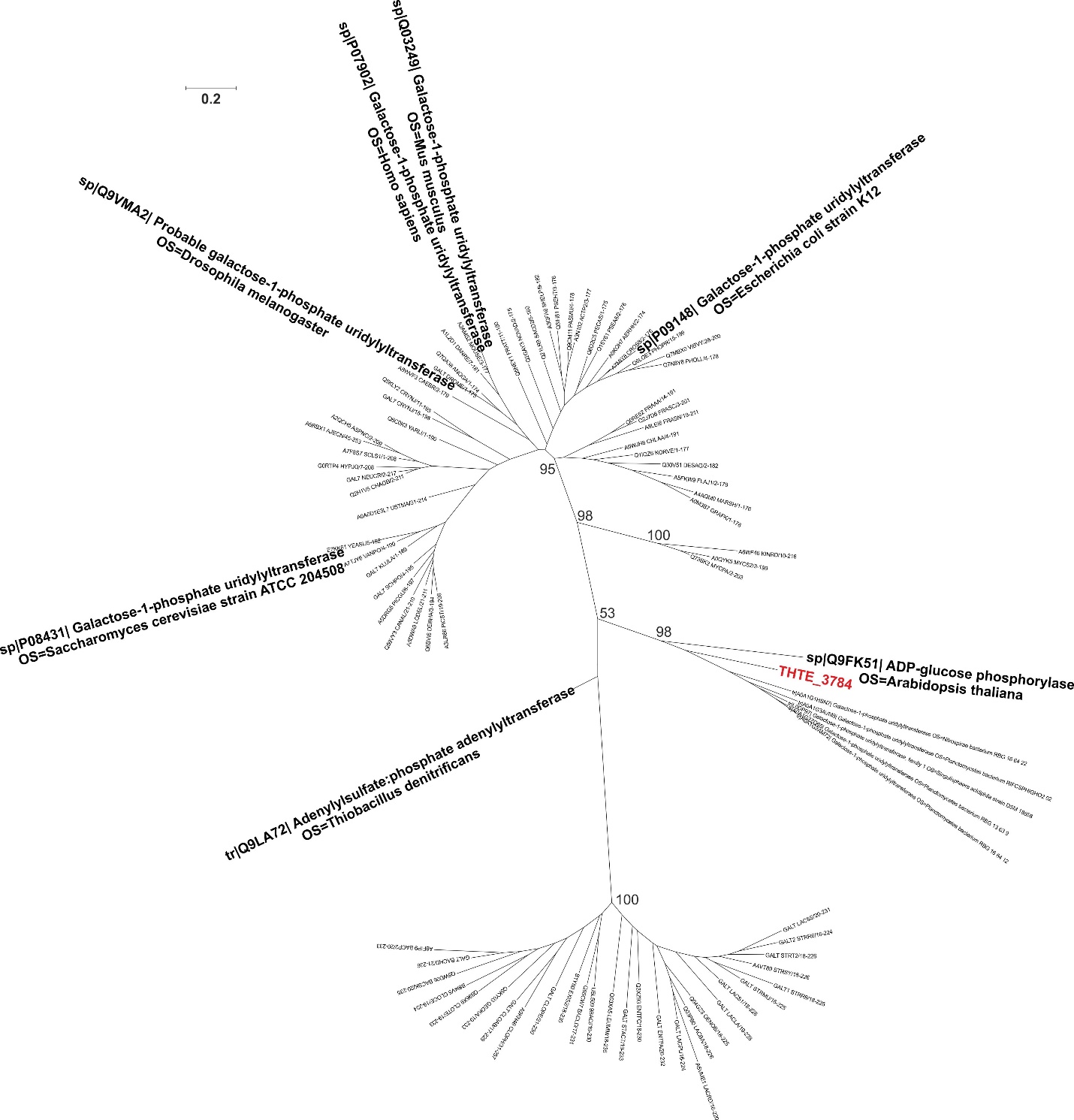


Supplementary Figure 1. Maximum likelyhood phylogenetic analysis of predicted galactose-1-phosphate uridyltransferase family proteins. *T. terrifontis* R1 enzymes are in red, biochemically characterized enzymes are in bold.


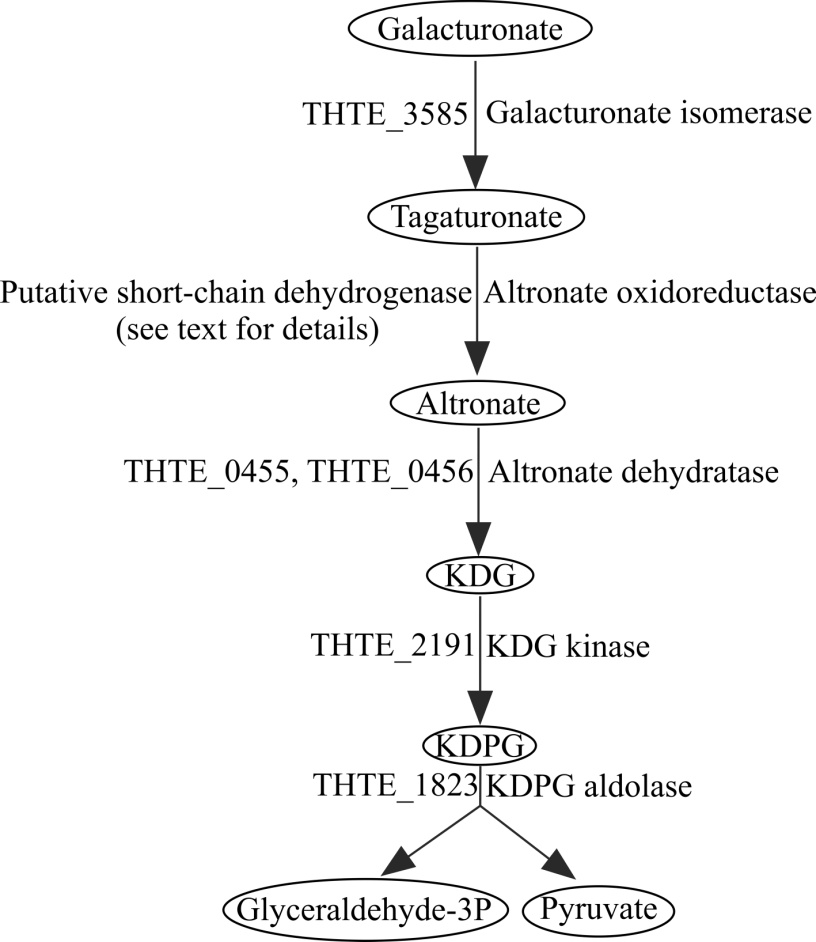


Supplementary Figure 2. *T. terrifontis* R1 Galacturonate degradation pathway.


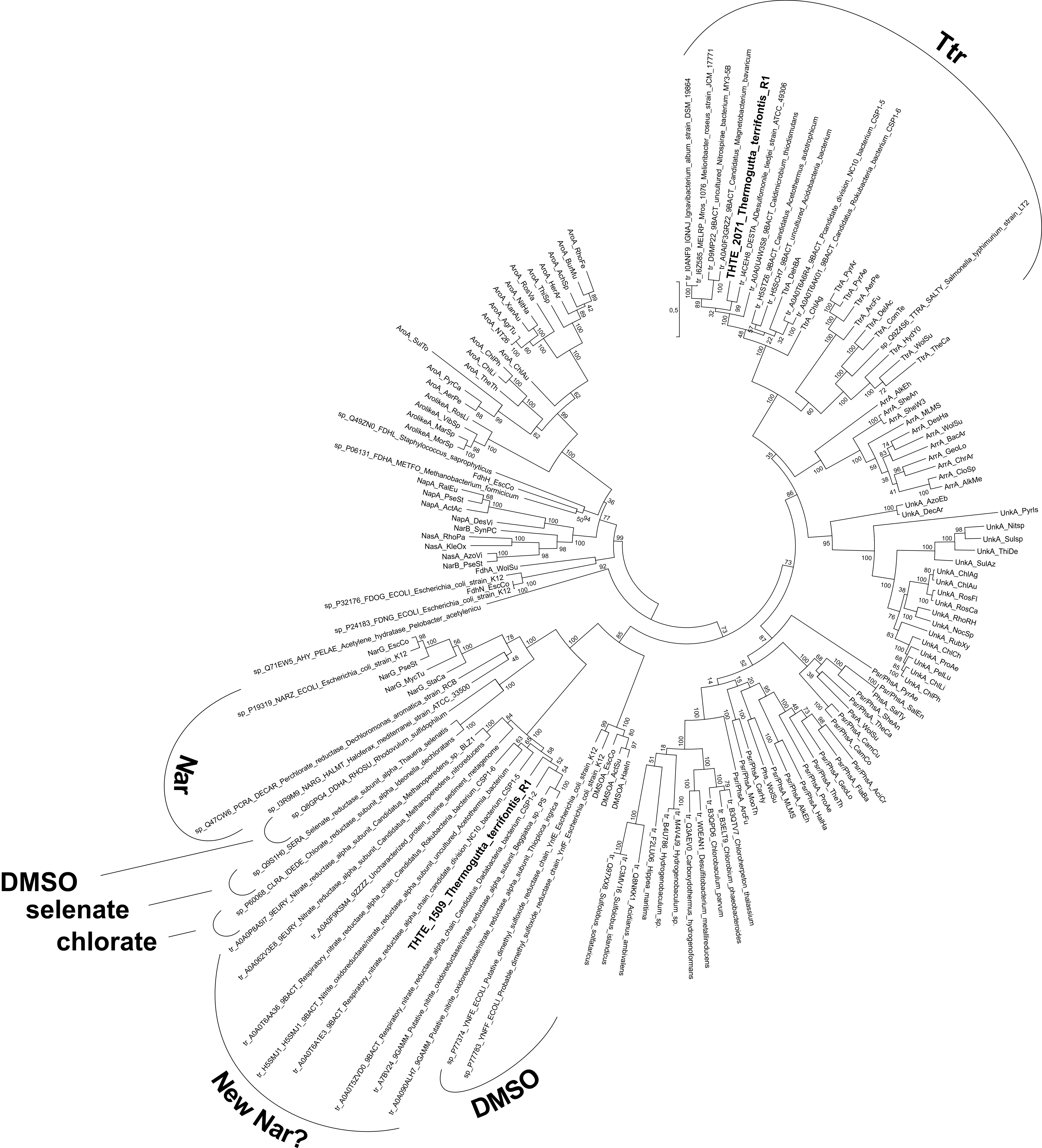


Supplementary Figure 3. *T. terrifontis* R1 molybdopterin oxidoreductases subunits A Maximum Likelihood phylogenetic analysis. The dataset and algorithm were the same as in Sorokin et al., 2016 with minor modifications. *T. terrifontis* enzymes are in bold. DMSO, dimethylsulfoxide reductases; Nar, membrane-bound nitrate reductases; Ttr, tetrathionate reductases.


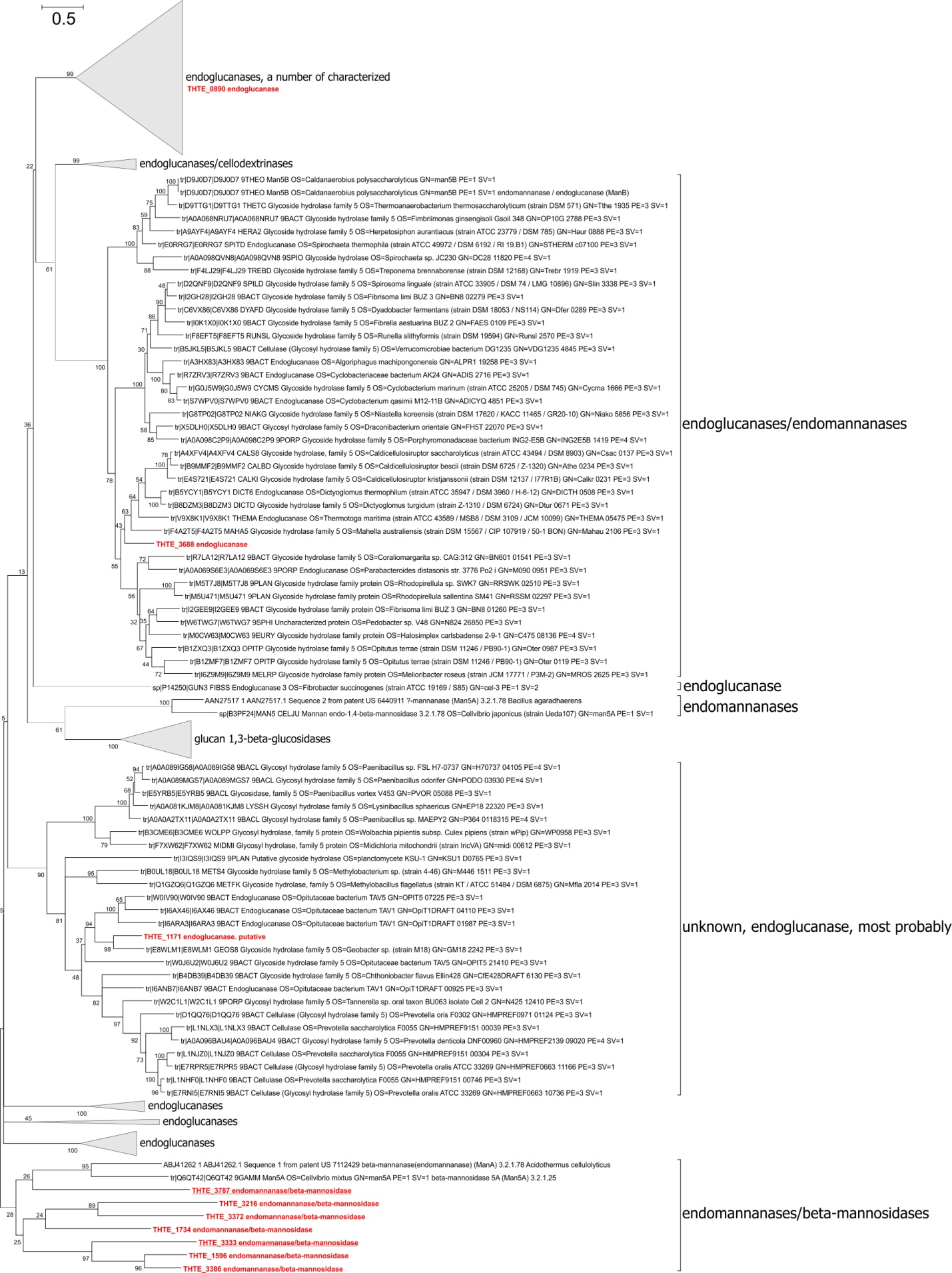


Supplementary Figure 4. Maximum likelyhood phylogenetic analysis of *T. terrifontis* R1 GH5 glycosidases. *T. terrifontis* enzymes are in red, up-regulated enzymes underlined.


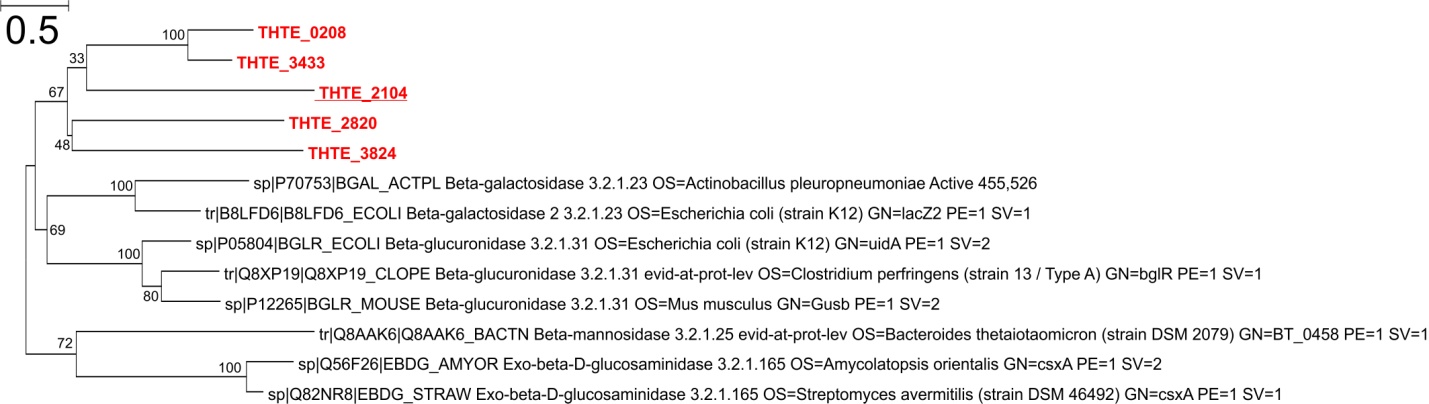


Supplementary Figure 5. Maximum likelyhood phylogenetic analysis of GH2 glycosidases. *T. terrifontis* R1 enzymes are in red, up-regulated enzymes underlined.
